# Semi-supervised learning framework for oil and gas pipeline failure detection

**DOI:** 10.1038/s41598-022-16830-y

**Published:** 2022-08-12

**Authors:** Mohammad H. Alobaidi, Mohamed A. Meguid, Tarek Zayed

**Affiliations:** 1 Department of Civil Engineering, McGill University, 817 Sherbrooke Street West, Montréal, QC H3A 0C3 Canada; 2 Department of Building and Real Estate (BRE), Hong Kong Polytechnic University, Kowloon, Hong Kong

**Keywords:** Energy science and technology, Energy grids and networks, Engineering, Civil engineering, Energy infrastructure

## Abstract

Quantifying failure events of oil and gas pipelines in real- or near-real-time facilitates a faster and more appropriate response plan. Developing a data-driven pipeline failure assessment model, however, faces a major challenge; failure history, in the form of incident reports, suffers from limited and missing information, making it difficult to incorporate a persistent input configuration to a supervised machine learning model. The literature falls short on the development of appropriate solutions to utilize incomplete databases and incident reports in the pipeline failure problem. This work proposes a semi-supervised machine learning framework which mines existing oil and gas pipeline failure databases. The proposed cluster-impute-classify (CIC) approach maps a relevant subset of the failure databases through which missing information in the incident report is reconstructed. A classifier is then trained on the fly to learn the functional relationship between the descriptors from a diverse feature set. The proposed approach, presented within an ensemble learning architecture, is easily scalable to various pipeline failure databases. The results show up to 91% detection accuracy and stable generalization ability against increased rate of missing information.

## Introduction

The petroleum industry comprises different stages of exploration, drilling, production and transmission. Oil and gas are the main fuel source of many industries and manufacturers. The oil and gas industries are no different from other fields in the challenges of meeting the efficiency and environmental regulations required nowadays. A resilient and functional transport system is required to maintain the continuous production and distribution of fuel. Although pipelines have the lowest accident rates compared to railways and other transportation means, failure in pipelines have catastrophic consequences, especially on the environment. The convoluted network through which the transmission of such product is made requires monitoring and maintenance^1^. Pipeline failure databases can then be designed to comprise operational conditions of one pipeline network at the time of the incident to aid in better understanding failure events and improving the critical energy infrastructure. Pipeline failure databases can also be constructed by simply pooling all failure incidents of various pipeline networks covering a wide region and do not share the same topology or operational characteristics. The latter are more commonly available as they do not require sophisticated monitoring and frequent inspection to populate such database with time-dependent explanatories^2^.

The advancement in digital technology plays a major role in enhancing the operation and modeling of oil and gas pipeline systems. Such technology can take advantage of pipeline operational data, comprising different measurements of pipeline network conditions, which requires development of robust clustering and analysis models to produce fast meaningful inferences. A complete data analysis of the available measurements and incident reports can be used in determining failure location and segments in the system^2^. This approach is challenged by the large amount of available data to generate a proper failure assessment model of the pipeline's network.

Complex chains of operation and networks can utilize the fast and robust decision-making frameworks recently proposed in the broad literature. For example, upgrading the pipeline's connection to a smart network can be done by the deployment of sensors across the system and using Inflow Control Valves (ICV) or Inflow Control Devices (ICD). While this improves the control and decision-making strategy of the system, the massive size and maturity of existing pipeline networks make digitalizing and information exchange across the system very costly^3–5^. The focus of many research studies is therefore shifted toward the efficiency of the data extraction processes rather than expensive complete upgrade of energy networks.

An important branch of data-driven modeling of pipeline networks is related to failure assessment. The Conservation of Clean Air and Water in Europe (CONCAWE) has been established to carry out research on the environmental impact related to the oil industry. CONCAWE associated the failures in the oil pipelines to causes related to operational and mechanical malfunctions, corrosions, and natural hazards, in addition to third-party activity^6^. These failure types are then adopted in vast majority of research in the field.

To gather intelligence on drivers to failure, inspection techniques have been applied to discover pipeline anomalies and flaws without shutting down production. Such approaches can be in the form of safety assessment frameworks for oil and gas pipelines which utilize a probabilistic modeling of a predetermined set of failure causing features^7^. This group of techniques is devised to increase the success of assessing pipeline health while managing to decrease inspection frequency and, hence, all associated costs. Nevertheless, pipeline failure events are prone to occur as the associated uncertainty of the driving factors cannot be fully treated^8^. In addition, maintenance of pipeline networks prompts continuous monitoring and inspection. Different inspection techniques have been industrialized in the last decade such as ultrasound and magnetic flux leakage. These techniques have been developed to detect anomalies in the pipelines^9–11^. However, considering the large scale of the pipeline network, these practices are, currently, impractical and entail a lot of time to conduct. The different geographical locations and ambient conditions make these assessment techniques and corresponding failure-causing anomaly detection models customized to failure types, system layout, and subjective to the site or operational engineer. To this extent, it is clear that more advanced failure detection techniques are required to monitor the safety of the pipelines and detect the type and location of failure to develop faster and better decision-making practices^12^.

### Machine learning and its application in pipeline failure assessment

The utilization of artificial intelligence (AI) allows solutions which circumvent the tradeoff between the complex physical system and its model representation. These solutions in return provide higher controllability and predictability of the system. The main subset of artificial intelligence relevant to this problem is machine learning, which can be further divided into three broad categories of modeling frameworks, supervised learning, unsupervised learning, and semi-supervised learning. In supervised learning, observations of the system are processed into inputs and outputs which are used to design and train a model that establishes a relationship between the system features and the predetermined outputs. This model can then be used to estimate the output of new instances which have the same feature space (or input configuration). In the case of unsupervised (clustering) learning techniques, information on the system features are used without an identified output. The features are exploited by the underlying learning process to create a number of clusters each of which contains similar instances. The similarity of instances is identified depending on the unsupervised learning strategy and its training approach. The resulting model is then used to map new instances into one of the predetermined clusters. Semi-supervised learning, the third category of machine learning, combines techniques from the previous two machine learning subsets to improve the performance or further exploit the available intelligence. In semi-supervised learning, the training of the input–output model is enhanced by the use of additional instances which are either missing its output or a subset of its features. To achieve this, an embedded clustering-like method is designed to learn from the incomplete instances and simultaneously make them available for the supervised training of models.

In the oil and gas industry, machine learning techniques can be applied to various types of large datasets which are generated by sensors deployed throughout the pipeline network (surface, subsurface) or from information collected passively. Machine learning techniques can therefore infer the behavior of the system from the available observations rather than relying on a physical model. Although such techniques have this advantage, the data related to big industries, such as oil and gas, are enormous and various. The data-driven modeling process and analysis of such pipeline systems may still require some understanding of the system behavior and computational resource.

Different supervised machine learning approaches are used in the literature as classification and regression solutions for specific pipeline system modeling objective; example of these are classification and regression tree (CART) artificial neural network (ANN), fuzzy logic (FL), response surface model (RSM), and support vector machine (SVM)^13^. For example, ANNs are utilized to predict the condition of three offshore oil and gas pipelines based on historical data specific to that pipeline network^14^. Available features, used to predict the discrete-type condition of the pipeline at a given time, include age, diameter, metal loss, and operating pressure. Another study develops multiple regression and ANN models to predict the cause of failure in onshore oil pipelines with a relatively similar predictor set as the former study^15^. The target variable represents failure types such as mechanical, operational, or corrosion based. While such models have been reported to predict the target variable with exceptional accuracy, the data based on which these studies are developed can be difficult to obtain by the scientific community and expensive to develop by service providers^2^. Consequently, pipeline-operating competitors in the oil and gas industry lack the collaboration required for machine learning research, as it heavily relies on the availability of data and the sharing of expertise gained from the studies conducted (open-source), which is not the practice of the oil and gas companies^12^.

### Incident-based databases for more useful data-driven solutions

A more useful, yet challenging to design, data-driven approach can be developed to exploit another type of databases which are publicly available and comprise incident reports for a wide range of pipeline networks. In general, such databases are mostly constructed by initial incident reports from the field, which then are refined as the incidents unfold. This failure information is recorded in large pipeline failure databases, many of which are made open access. Incident logs are mandatory upon any failure event and their collection is, hence, relatively cheaper and more suitable to both government regulation entities and service providers^7^.

Typically, engineers rely on incident reports to address failure events, which would take time assessing, evaluating, and responding to the event. The inefficient process in determining the damaged pipes and responding to the failure adequately increases the environmental and financial losses. Human errors in reporting and subjective documentation of incidents are another crucial drawback in conventional practice and further jeopardize the response process. As a consequence, gathering historical failure information about pipeline incidents is a valuable mean of analyzing failure behavior and consequences. This also allows for developing different approaches which targets a more direct modeling of the relationship between failure factors and, for instance, magnitude of failure. Such modeling framework has the advantage of being scalable to a wide variety of more accessible energy pipeline information and publicly available databases^15^.

### Motivation behind the proposed approach

The question here is related to how we can utilize the incident reports in determining the cause of the failure event, prompting a more suitable and faster response. To do so, the detection approach should overcome potential limitations usually inherent in failure logs, such as missing information, which disrupt the design of a constant and informative feature space serving as the input to a supervised learning framework. The latter should be integrated in a machine learning approach to allow on-the-fly flexible intelligence-extraction and maximum utility of the available database. The problem, hence, demand semi-supervised learning techniques which are capable of not only cluster-then-classify (CTC) routines, but also a contiguous imputation stage. While the broad literature provides CTC examples, there has been no attempt to utilize CTC, or the required cluster-impute-classify (CIC) approaches in this field^16,17^. There is, however, no study to date, which develops a semi-supervised learning model nor CIC framework for the problem of interest. To this extent, this work develops a CIC framework which is able to accommodate the challenges in the problem of interest, all while producing a stable generalization ability with limited information, be it the size of the database or the degree of missing information in the incident report. The benefits of such framework are made two folds by its ability to adaptively learn over diverse feature set from different databases and autonomously impute important variables for a specific database application utilized by the relevant industry.

## Methods

**Figure 1:**
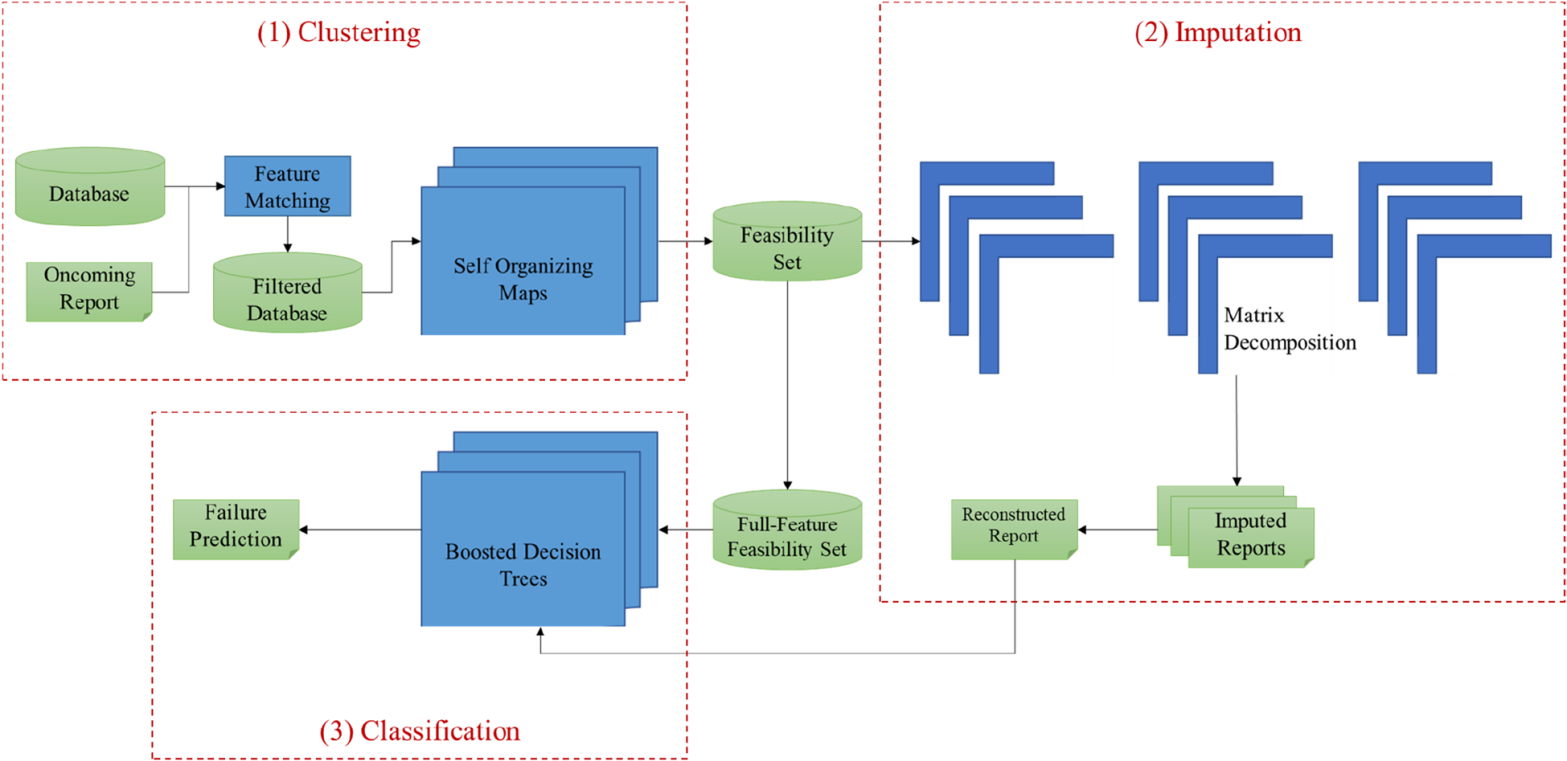
Proposed CIC framework for detecting pipeline failure events. The ensemble based semi-supervised approach is divided into: (1) Clustering, (2) Data Imputation, and (3) Classification. The separability between the three CIC stages allows for a more adaptive application and easier tuning of the models’ hyperparameters.

This work proposes a CIC framework within an ensemble architecture. The ensemble learning approach is designed to serve different phases of the CIC components, especially the imputation phase. Figure 1 depicts a visual overview of the proposed learning framework. The proposed approach belongs to the Cluster-Then-Classify family of methods under Inductive-type semi-supervised learning^18^.

The CIC model within the proposed framework comprises three primary phases, clustering, imputation, and classification. The CIC approach is manifested in an ensemble learning environment. The authors recognize that an optimal description of the proposed framework can be achieved by processing an input with respect to a training set. Suppose that we have a set of *N* observations, of which *l* is labeled, $D_{L} = \left( {x_{i} ,y_{i} } \right)_{i = 1}^{l}$, and a reminder of unlabeled instances, $D_{u} = \left( {x_{i} } \right)_{i = l + 1}^{N}$, where $x_{i}$ is the input from a predetermined feature space $X$, and $y_{i}$ is the output label (if available). The ultimate task is to quickly generate a model which identify the class $y^{*}$ of an unlabeled instance $x^{*}$.

### Stage 1: Clustering

First, the unlabeled instance may have missing features with respect to $X$. To reconstruct the missing information, we generate a set of *S* resamples, $\left( {D_{i} } \right)_{i = 1}^{S}$, from the combined set of $D_{u}$ and $D_{L}$, via a Bootstrap^19^, each of which has the same size *N* and is clustered in parallel as well as independent of each other. In this work, Self-Organizing Maps (SOMs) are utilized for the clustering phase. SOMs belong to a class of ANNs which involve a nonlinear projection of the input space to a low-dimensional output space^20^. The input instances are grouped into a predetermined number of clusters.

During training of the SOM, there is a competition between the output neurons to fire, which guides the clustering effort of input instances. Such output neurons are often called ‘winner-take-all’ units. The aim of competitive learning is to cluster the data. As it is the case with Hebbian-type learning networks, there is no labelling information passed to the network during its training, even if such information exists. Instead, the SOM must self-organize on the basis of the structure of the input data. The main idea behind competitive learning is to find a winning unit and update its weights to make it more likely to activate if similar inputs are introduced to the network. Research on SOM is continuously evolving, and extensive work is available in the literature^21^.

Only the common features between the resampled subset and the test instance will, obviously, be utilized at this stage. From validation, SOMs with 1 × 3 topology have been determined optimal for the considered application. In addition, one of the clusters, to which $x^{*}$ belongs, is used to identify the corresponding observations which will be used in the imputation phase. It is important to note that this process is repeated *S* times to generate the corresponding subsets for the imputation phase.

### Stage 2: Matrix decomposition and imputation

At this point, we have *S* sets of observations coming from the individual SOMs. Each $D_{i}$ is then used to construct a feasibility set $X_{i}$, with effective size $n_{i}$, based on which a decomposition model is set. $X_{i}$ includes $x^{*}$ but may not comprise all of the clustered observations in the corresponding set $D_{i}$, hence the term effective size. This approach is determined by the authors to control for parameter dilation and strengthen the identity of the estimate^22^. In addition, a scaled version of the feasibility set is used in the factorization problem, as a positive matrix factorization is important for improved estimation, and wide-enough margins between the categorical entries further facilitates the convergence^23^. The decomposition model is in the form:

1 $$X_{i} \cong I_{i} \cdot \mathop \sum \limits_{j = 1}^{{K_{i} }} V_{j}^{i} \cdot W_{j}^{iT}$$

where $V_{j}^{i} \in R^{{n_{i} \times 1}}$ and $W_{j}^{i} \in R^{{n_{i} \times 1}}$ $\left( {V_{j}^{i} , W_{j}^{i} \ge 0; i = 1,2, \ldots ,S;j = 1,2, \ldots ,K_{i} } \right)$ are the positive latent vectors partly representing the reduction of the feasibility set generalizing over $D_{i}$, and $K_{i}$ is the number of corresponding vector pairs. In order to account for missing information in the feasibility set, the indicator function $I_{i}$ is utilized, where it is set to zero when incident log entries are detected, and one otherwise.

In addition, the authors treat $K_{i}$ as a hyperparameter, where the proposed learning framework is designed to autonomously identify optimal number of latent vector pairs for each dispatch process, and independently for each sub-ensemble feasibility set $i$. The loss function that we need to minimize is as follows^24^:

2 $${\mathcal{L}}_{i} = \frac{1}{2}\mathop \sum \limits_{t = 1}^{{n_{i} }} I_{i} \left( t \right) \cdot \left( {X_{i} \left( t \right) - \mathop \sum \limits_{j = 1}^{{K_{i} }} V_{j}^{i} \left( t \right) \cdot W_{j}^{i} \left( t \right)^{T} } \right)^{2} + \frac{{{\uplambda }_{i} }}{2}\left( {\mathop \sum \limits_{j = 1}^{{K_{i} }} ||V_{j}^{i} ||_{F}^{2}  + \mathop \sum \limits_{j = 1}^{{K_{i} }} ||W_{j}^{i} ||_{F}^{2}} \right)$$

where || · || represents the Frobenius norm, λ is the regularization parameter, and *t* is the entry of incident log ($t = 1, 2, \ldots , n_{i}$). The loss function promotes the constrained probability mass function (PMF) identity of the factorization and drives the factors to take near-sparse form in the latent vector arrangements. Once all the sub-ensemble latent vector parameters are estimated, the missing information from the target observation is then computed as follows:

3 $$\hat{x}^{*} = \frac{1}{S}\mathop \sum \limits_{i = 1}^{S} \mathop \sum \limits_{j = 1}^{{K_{i} }} V\left( {t = t^{*} } \right)_{j}^{i} \cdot W\left( {t = t^{*} } \right)_{j}^{iT}$$

The authors utilize Alternate Least-Squares Algorithm (ALS) to find appropriate solution to the latent vectors. At this point, the oncoming incident log has been preprocessed and any important missing information is imputed. The target observation is retained in a master subset which comprises relevant incidents from all the *S* feasibility sets as well as other observations not related to the target by SOMs. This further promotes diversity in the master set and is expected to improve the generalization ability of the final stage learner. The master set is referred to as the full-feature feasibility set in Fig. 1.

### Stage 3: Classification

The objective at this stage is to construct a classifier which is able to identify pipeline failure types from oncoming incident reports for which a number of feasibility sets has been constructed in order to perform the necessary imputation and prepare the reconstructed, complete observation. The classifier is obviously required to have reliable generalization ability and, equally important, easily deployed for industry application with limited computational resource. While it has been showed that state-of-the-art deep learning models provide sufficient performance in applications comprising sufficient intelligence, problems with limited feature set pose similar performance stability issues as other machine learning models^25^. Under such conditions, ensemble learning provides a suitable solution. Ensemble learning, a recent advancement in machine learning, deals with generating multiple detection models which are trained using subsets (resamples) of the database. The outputs from each individual member of the ensemble model are then fused to make a combined inference. Ensemble learning is shown to relatively produce models with diminishing uncertainty and more stable generalization ability^26^. The broad literature is continuously providing work discussing the effectiveness of ensemble learning^27–30^.

Generally, for a learning framework to be considered an ensemble learning model, it should undergo three fundamental steps. The first step is resampling^31^, which consists of generating a number of subsets (resamples) from the available set of data. The second step is sub-ensemble model generation and training; the individual models, or sub-ensemble models, are tuned (trained and validated) using their corresponding resamples. The third step is ensemble integration, which merges the output of the individual models to produce a global (ensemble) output. Ensemble learning frameworks are divided into two broad clusters, homogeneous ensembles and non-homogeneous ensembles^32–34^. In homogeneous ensemble learning frameworks, sub-ensemble models are given their resamples via the same resampling technique, and they stem from the same machine learning model, where the ensemble model has only one integration technique^35,36^. Non-homogeneous ensemble learning frameworks do not adhere to one or more characteristics of homogenous ensembles, but maintain the three fundamental steps of ensemble learning^34,36^.

In this work, boosted decision tree ensembles are utilized to serve as the classifier stage in the proposed framework^37,38^. This process starts with unweighted events and build a CART. If a training event is misclassified, then the weight of that event is increased (boosted). A second tree is built using the new weights, no longer equal. Again, misclassified events have their weights boosted and the procedure is repeated. Typically, one may build hundred of trees this way, comprising the ensemble size of the boosted tree model (number of individual CARTs in the ensemble). The RUSBoost Algorithm is utilized to generate and train the boosted tree ensemble model^38^. The choice of CARTs as a building block of the developed ensemble model is due to the nature of the feature space accompanying this problem. The mixture of continuous, ordinal and other categorical features prompts the need for CARTs, as they allow for efficient space segmentation with mixed feature sets. Also, the use of boosted trees as and alternative to ensemble ANNs has been shown to sometimes improve the generalization ability in the literature^38,39^.

## Results and discussion

### Database

The database of the pipeline and hazardous materials safety administration (PHMSA) of the US Department of transportation, spanning the years 2004 to 2021, is used in this work^40^. PHMSA database contains information on various types of pipelines and is divided according to the classification of pipelines. Pipeline divisions are mostly gas transmission/gathering, gas distribution as well as hazardous liquid pipelines. In each class, general records of pipeline failure incidents are collected along with incident locations, operators, pipeline characteristics, failure magnitude and other standard incident markers.

Furthermore, the database contains a detailed description about many of the failure incidents, which pertains to the cause of the failure, an estimate of associated cost, environmental consequences of the incident, as well as the overall inspections that have been done during the pipeline’s operation. Installation year of pipelines, date of preliminary incident report, maximum allowable and operating pressure, and pipeline’s material strength are also recorded. While there are various features, most are recorded after the preliminary incident report is issued, and for reliable simulation these are not used here. Instead, this work relies on features based on information which is assumed to accompany an oncoming alarm or report, with some form of supervisory control and data acquisition (SCADA) system which provides fast access to basic pipeline information (both characteristic- and operation-type features).

Most energy pipelines in the United States are connected to a SCADA system and so this assumption is reasonable. However, for reliable undertaking of the diverse situations an operator may face when receiving an incident log, the study should not assume that all potential features will be available every time an incident is logged. Hence, while a feature set is assimilated from the PHMSA database, the study will assess the generalized framework over incidents with missing information, up to half of the preset feature space. Nineteen features, twelve continuous and seven categorical, are identified in this work and reported in Table 1. The target feature in this work is the type of failure, be it a leakage, a mechanical puncture, or a devastating fracture in the pipeline. These failure types are assumed to constitute the magnitude of the incident and prompt a reasonable estimate to post-accident consequences.

**Table 1:** Descriptive statistics of the utilized features for detecting pipeline failures.

Feature	Mean	Median	Minimum	Maximum	Standard Deviation
Unintentional release (MCF)	24,318.20	4,000.00	0.00	585,457.00	57,576.27
Intentional release (MCF)	4,354.93	205.00	0.00	89,152.00	10,084.19
Cover depth (ft)	51.00	42.00	0.00	336.00	48.89
Pipe diameter (in)	17.40	16.00	2.00	42.00	9.20
Pipe thickness (in)	0.28	0.25	0.13	1.00	0.09
Pipe strength (psi)	45,839.39	46,000.00	29.00	80,000.00	11,802.71
Potential impact radius (ft)	352.18	335.00	23.00	1,155.00	209.27
Operational pressure (psi)	656.91	690.50	1.50	2,689.00	317.75
Max allowable pressure (psi)	814.85	810.00	120.00	3,255.00	358.50
Potential isolated segment (ft)	59,425.05	48,014.50	1.00	801,150.00	79,836.08
Pipe age (years)	52.70	54.00	0.00	96.00	17.66
Impact area (in^2^)	3,273.60	1.67	0.00	114,022.50	12,195.38
Prompted shutdown (1:yes, 2:no)	1.91	2.00	1.00	2.00	0.29
Ignition (1:yes, 2:no)	1.15	1.00	1.00	2.00	0.36
Crossing (1:yes, 2:no)	1.12	1.00	1.00	2.00	0.32
Pipe coating type (multiple)	–	–	–	–	–
Location class (multiple)	–	–	–	–	–
Previous inspection (1:yes, 2:no)	1.56	2.00	1.00	2.00	0.50
SCADA anomaly flag (1:yes, 2:no)	1.20	1.00	1.00	2.00	0.40

Observations with partially available information are included in the dataset used to generate the statistics. The training and testing datasets are normalized before proceeding to the proposed CIC framework.

### Clustering and data imputation stages

The proposed CIC framework augments the investigation of optimal clustering configuration with the proposed factorization technique. Clustering is not only used to improve the final classification performance as in CTC frameworks, but also to guide the selection of useful information sets for efficient factorization in order to impute the missing information in the incident log. The explanatory validation study, carried out in this work, shows that a SOM with 3 × 1 topology is the most suitable for the utilized database. This may differ for other databases, and so the proposed framework must be applied accordingly; an explanatory validation study as well as a feature selection study must be carried out a priori for optimal results.

The robustness of the utilized framework should be tested against different aspects in order to ensure a more comprehensive assessment. At stages 1 and 2, these aspects are the amount of potentially missing information as well as the required number of incident observations for stable performance. The classifier is then tested at the critical thresholds in both aspects for reliable testing. As there any combination of the considered features can be potentially missing, a universal metric is required to evaluate the overall data reconstruction effort regardless of the number of missing variables per incident. A reasonable measure to summarize the estimation of all imputed features is considered to be the Pearson Correlation Coefficient (*R*^2^) here. In addition, the overall deviation error (taken from the first norm in the proposed factorization technique) between the observed and reconstructed feasibility sets can be reported along with the computational cost. These two additional metrics may be used to decide the critical fraction of missing information beyond which the proposed approach cannot handle, remain high, or be computationally expensive. To identify the critical fraction of missing features, a *R*^2^ of 0.9 is set as the minimum accepted overall fit.

**Figure 2:**
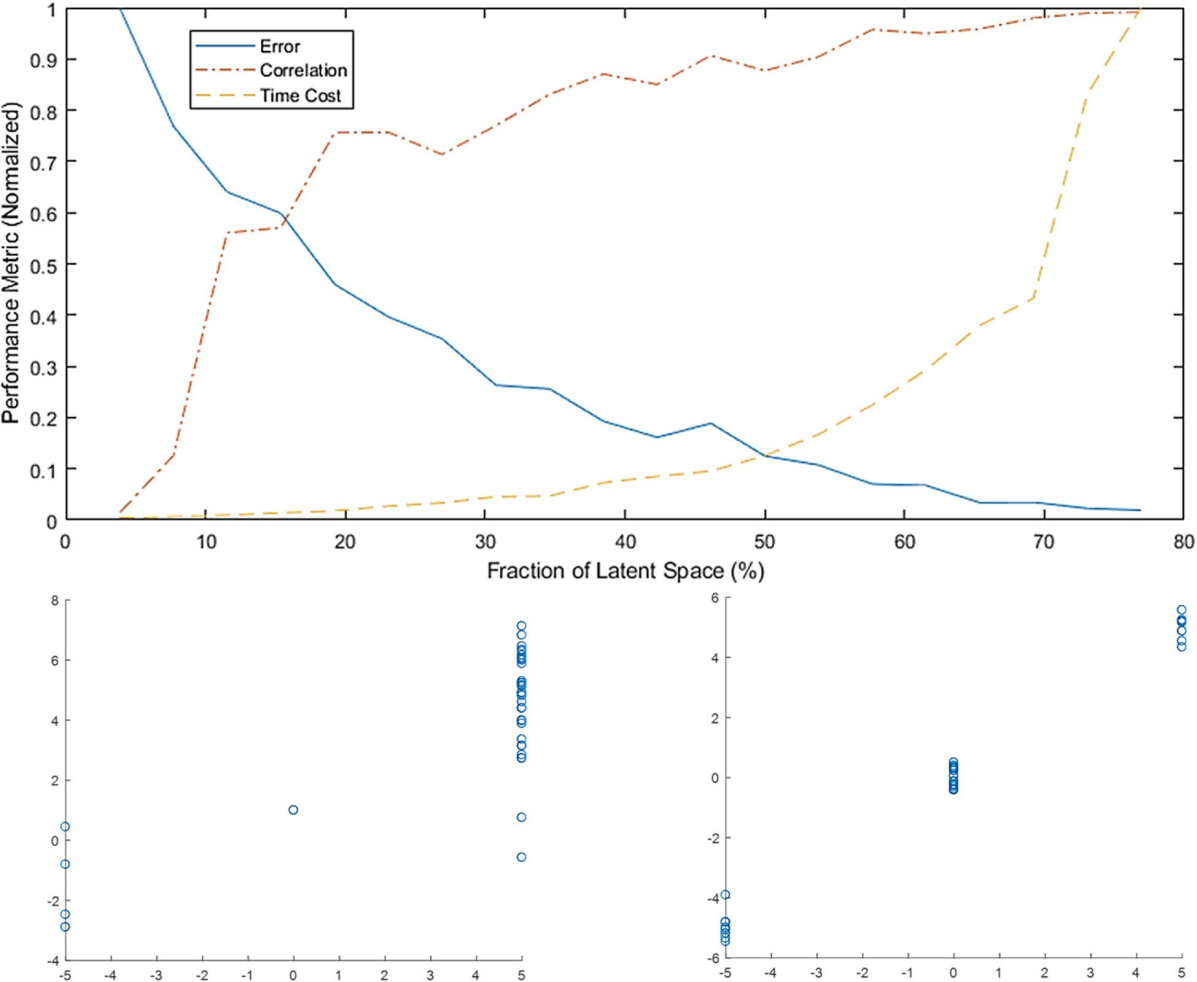
Reconstruction performance (training) of the proposed ensemble (size 5) decomposition approach at the critical feasibility set size and critical missing information size (50%). Top: fitness, error and computational cost versus the laten space size. The validation results demonstrate the stability of the CIC framework and validate the optimization efficiency of the presented tensor decomposition approach. Both error and computational cost are normalized with respect to their respective highest values in the simulations. Bottom: comparison between the reconstruction performance without SOMs (left) and with SOM (right) of a categorical feature with three classes. A five-unit difference is used to allow appropriate separability between the classes. In the simulations without SOMs, the middle class (value of zero) observations are stacked on top of each other, showing only one visible value. This is due to the influence of its size (the majority of the observations are in the middle class, prompting unfavorable reconstruction outcome).

Figure 2 depicts the performance of the proposed cluster-then-impute approach with respect to missing information. In order to simplify the analysis, without compromising the stress-testing of the approach, the experiment also pairs the number of utilized features in the factorization problem with the number of missing features. The approach is able to maintain adequate reconstruction quality with less than half of the features being available. Also, a near-perfect reconstruction is obtained without using all available features in the feasibility set (75% of the features). This does indicate that a reasonable interrelationship exists between the features, but not to a limit of redundancy, as the features represent physical-, operation- and incident-related explanatories.

It should be noted that no certain feature is preset to be missing, and a randomized combination of features are selected for each of simulations in the figure. While the cluster-then-impute approach has an ensemble of size five in this study (different from the ensemble learner’s size at stage (3), increasing the ensemble size is expected to further decrease the threshold fraction of missing features but increase the computational cost at the same time.

The figure also shows the importance of the clustering stage. Without a refined feasibility set, both the continuous and categorical features (the latter being hardest to impute due to their corresponding nature) will exhibit erratic and undesired factorization. The shown categorical variable is the class location, and without clustering, the factorization’s inability to identify consistent latent space segmentation is translated as the misclassification of edge categories onto the middle one (tending toward the mean which is zero here). However, when an ensemble SOMs are utilized, a more interrelated feasibility set is obtained, and the laten space identification is more useful and adequate reconstruction of the categorical feature is obtained.

**Figure 3:**
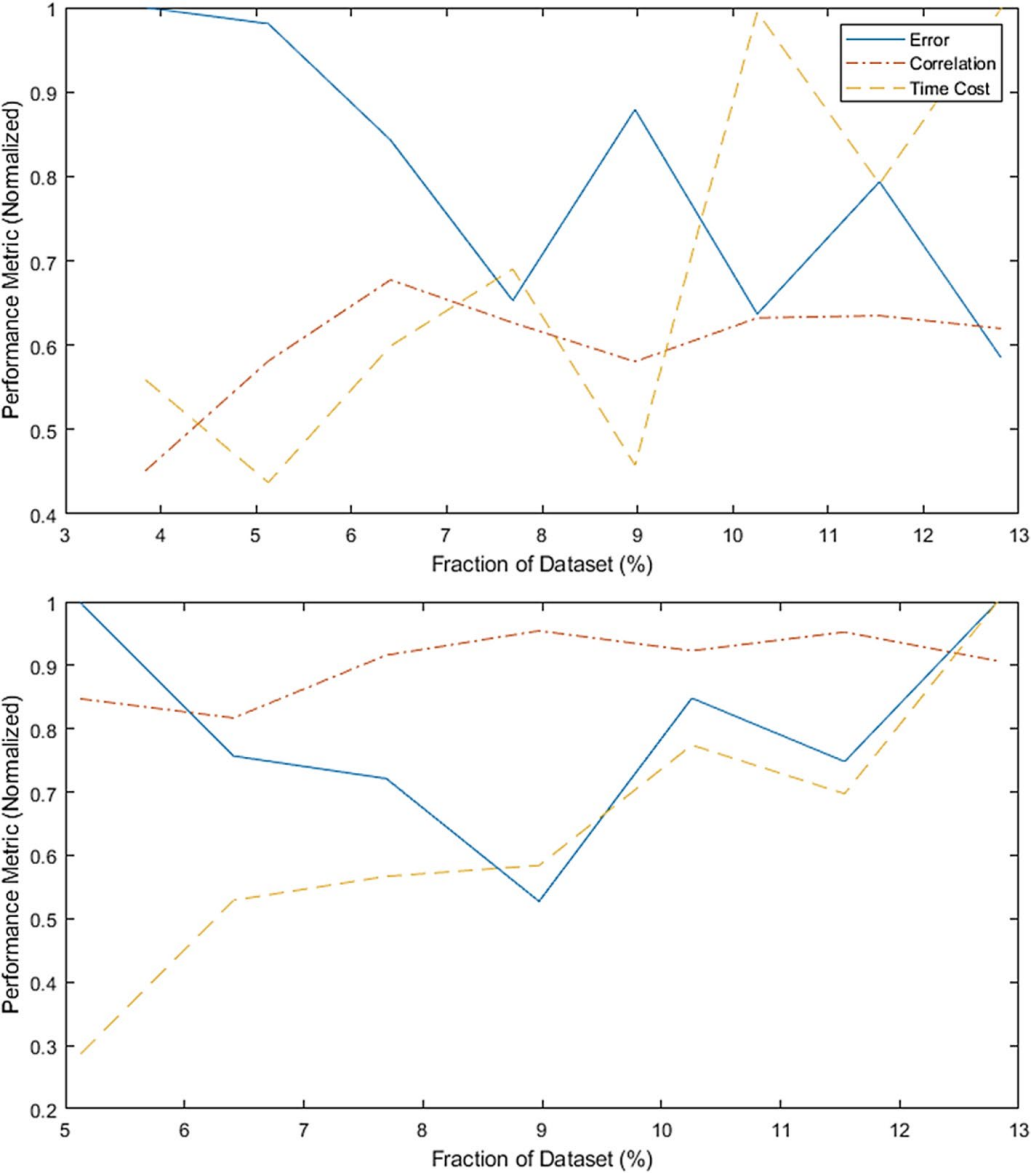
Reconstruction performance of matrix decomposition without clustering (top) versus that of the proposed ensemble (size 5) approach with ensemble SOM clustering layers (bottom) with respect of available information and missing information (matched). The simulations are carried out with respect to the critical latent space size (50%). Fitness, error and computational cost versus the latent space size are reported. Both error and computational cost are normalized with respect to their respective highest values in the simulations. The validation results are intended to clearly show that a search for an optimal sample size of the master set is significant for an improved CIC performance. This is shown by the U-shaped performance in the bottom sub-figure.

Figure 3 presents the performance of the two stages with respect to the number of observations, from the feasibility set. These simulations are carried out at the critical fraction of missing features (set at 50% missing information). When no clustering is utilized, the factorization approach produces low overall fitting performance. Also, it is clear that the error, while initially high, oscillates with increasing sample size. At lower number of paired observations, there is a low likelihood of having truly relevant observations. As the factorized matrix size grows, more relevant instances is expected to be present, but also more nonrelevant observations (with the latter being twice as likely since the optimal SOM identifies three clusters). Consequently, the error will not stabilize, and the fitting metric will show an overall poor performance.

On the other hand, the clustering stage identifies a more related feasibility set which is then fed into the decomposition approach. The optimal SOM, as well as the nature of the properly selected features in the training set, prompt a stable and acceptable information reconstruction performance. Note here that the figures, while plotted with percentage fraction of the feasibility set, are based on different numbers of observations. This is because the SOM will propose three clusters, one of which is used in the factorization, which is inherently smaller than the training set.

As a consequence, the clustering approach is shown to significantly improve the factorization stage despite the decrease in the final sample size. In this validation phase, a randomly selected set of observations are used here as a testing set to track the performance in both No-SOM and SOM scenarios. However, the same ensemble size (5) is used, where a random resample is drawn from the feasibility set each time a cluster-then-impute model is constructed.

### Classification stage

To further demonstrate the reason behind using boosted trees, different machine learning models are investigated in this work. Table 2 summarizes the performance of the utilized models. The boosted trees ensemble model shows the best testing performance. This is in line with the expected behavior of the utilized models, given their learning approach and the nature of the available feature space. It is also worth noting that all CART-based models show desirable performance, which further validates the choice of this class of models.

**Table 2:** Validation performance of utilized machine learning models.

Model*	Class	Accuracy	False positive	False negative	Overall accuracy
KNN	1	56.4	41.3	43.6	59.0
	2	53.8	45.5	46.2	
	3	53.8	36.6	33.3	
Naïve bayes	1	74.3	25.7	33.3	72.2
	2	58.1	41.9	30.8	
	3	88.7	11.3	19.2	
SVM	1	78.2	21.8	21.8	76.1
	2	73.1	36.0	26.9	
	3	76.9	10.4	23.1	
CART	1	96.2	3.8	2.6	88.9
	2	86.8	13.2	15.4	
	3	83.5	16.5	15.4	
ANN	1	88.0	12.0	12.0	88.6
	2	82.5	17.5	19.5	
	3	**94.0**	**6.0**	**4.1**	
Boosted trees	1	**97.4**	**3.8**	**2.6**	**91.0**
	2	**91.0**	**12.3**	**9.0**	
	3	84.6	10.8	15.4	

All models' performance metrics are evaluated based on a five-fold cross validation study.

*The exact computational time depends on the configuration of the parallel computing environment. In this case, local parallelization is used, 4 cores sharing 24 GB. The average computation cost to train the complete CIC framework and assess a failure event from a given incident report is ~ 8 min. This is based on the optimal CIC configuration and using the aforementioned computational settings.

Best performance values are in [**bold**].

**Figure 4:**
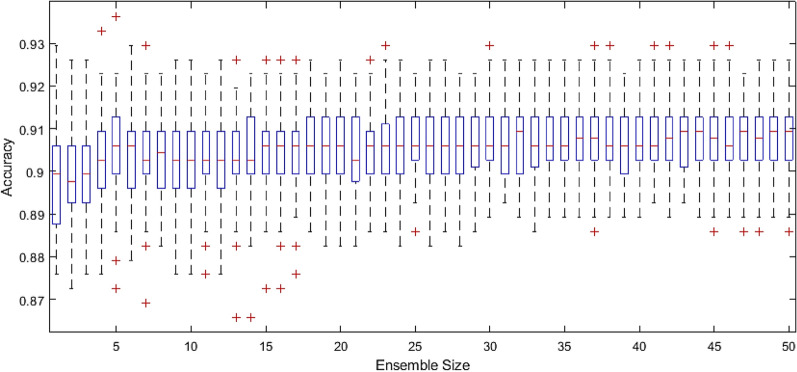
Generalization ability of the proposed model (complete CIC) based on the boosted trees ensemble versus the ensemble size. the boxplots are based on Monte Carlo simulations of complete re-runs of the models, not just error-scatters of testing set of an individual simulation. In each simulation, the CI stages are configured based on the critical feasibility set size, latent space size and missing information proportion.

In addition, it is important to investigate the optimal size of the ensemble model so that the tradeoff between the model’s generalization ability and computational cost is balanced. Figure 4 depicts the increasing generalization ability, and diminishing uncertainty, with the increase of the ensemble size. This behavior is expected as per the diversity in learning theory of ensembles. In addition, at higher ensemble size, the models exhibit a relatively more stable performance which is required for reliable deployment of the proposed approach. However, while the usual behavior of larger ensemble models dictates a more noticeable increase in performance, the boosted trees model’s performance saturates beyond a certain ensemble size. This behavior can be regarded to the fact that the factorization stage may incur a performance ceiling due to the low-rank decomposition. While this observation may prove undesirable at certain situations, the proposed framework is able to successfully provide a good detection capability (91% overall classification accuracy), which is the most important outcome to such modeling frameworks.

**Figure 5:**
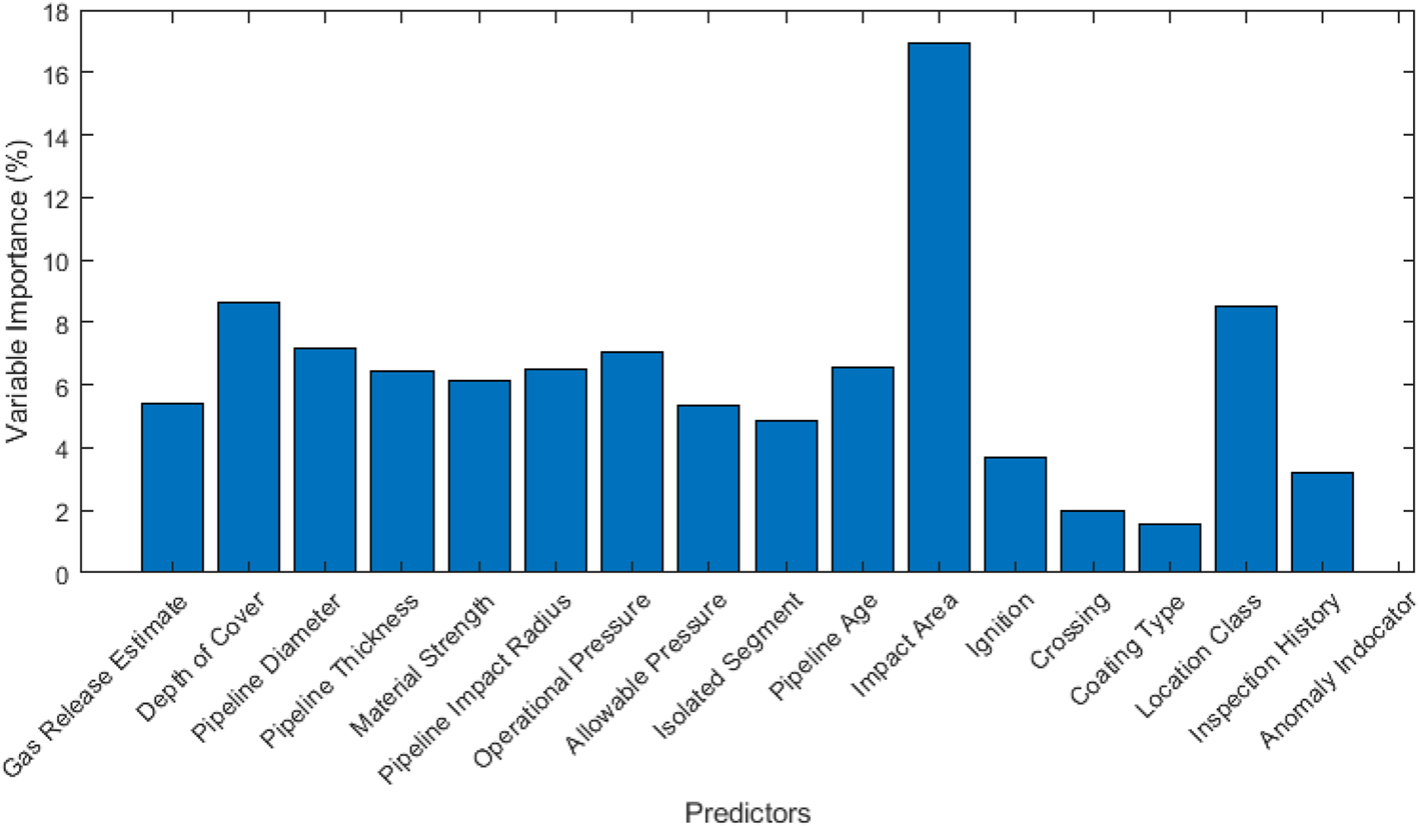
Variable importance of the top features in the study. The results are based on analyzing the deterioration of CART models with respect to removing one of the variables and reporting the relative performance of the model based on all experiments. Notice that the built-in anomaly indicator in the SCADA system (when available) does not provide useful information to the machine learning model, as it is often not reliable.

A final explanatory study is also presented in this work. Figure 5 shows the variable importance of a subset of the utilized features in the training set. A balanced feature importance profile is shown among the significant explanatories. The SCADA based categorical feature, while has minimal importance to the boosted trees model’s generalization ability, is shown to validate the importance of the proposed framework. (i.e. an alarm may indicate any of the three failure types equally likely). The variable importance study can be used to further tune the feature selection process. Moreover, it can be used to improve the proposed cluster-then-impute stages of the modeling framework by modifying the decomposition’s objective function. In other words, the latent space construction phase can be weighted with respect to the corresponding importance of each reconstructed feature, giving priority to more important features from a classifier perspective. This is one objective of the future work in this field which the authors aim to pursue.

## Conclusions

The proposed semi-supervised framework is able to detect the failure type of gas pipelines from raw databases with almost nonexistent filtering scale. The adaptive strategy can reconstruct missing information from failure reports regardless of the type or number of omitted features. The imputed feasibility set shows that clustering is very important to properly match the oncoming report with available history in the database. The ensemble learning approach has also resulted in improved stability with increasing ensemble size. This work is aimed to prompt industry and government entities to invest in developing robust and relatively cheap infrastructure monitoring approaches which can substantially reduce the consequences of oil and gas pipeline failure events. While the separability of the proposed CIC framework promotes computational efficiency and freedom of adapting the semi-supervised stages, this may cause a larger permutations of validation studies to find optimal configuration. Additional complexity in the training phase may arise if, for example, the utilized database holds a limited feature set, and so future work should investigate such challenges under more detailed data-and-feature availability scenarios.

## Supplementary Information

Supplementary Information.

## Data Availability

Data used and generated in the study is included in the main manuscript as well as supplementary file.
